# The Various Roles of PEDF in Cancer

**DOI:** 10.3390/cancers16030510

**Published:** 2024-01-24

**Authors:** Mitra Elmi, Joshua H. Dass, Crispin R. Dass

**Affiliations:** 1 Curtin Medical School, Curtin University, Bentley, WA 6102, Australia; mitra.elmi@postgrad.curtin.edu.au (M.E.); joshua.dass@curtin.edu.au (J.H.D.); 2 Curtin Health Innovation Research Institute, Curtin Medical School, Curtin University, Bentley, WA 6102, Australia; 3 Sir Charles Gairdner Hospital, Nedlands, WA 6009, Australia

**Keywords:** cancer, tumour, oncology, PEDF, diagnosis

## Abstract

**Simple Summary:**

Pigment epithelium-derived factor (PEDF) is a versatile protein with potent effects against various cancers. It hinders cancer initiation and spread. This review highlights some of the molecular pathways involved in PEDF’s anticancer properties.

**Abstract:**

Pigment epithelium-derived factor (PEDF) is a natural immunomodulator, anti-inflammatory, anti-angiogenic, anti-tumour growth and anti-metastasis factor, which can enhance tumour response to PEDF but can also conversely have pro-cancerous effects. Inflammation is a major cause of cancer, and it has been proven that PEDF has anti-inflammatory properties. PEDF’s functional activity can be investigated through measuring metastatic and metabolic biomarkers that will be discussed in this review.

## 1. Introduction

### 1.1. PEDF Structure

The protein pigment epithelium-derived factor (PEDF), identified in 1987 [1], is a 46.3 to 50 kDa serine protease inhibitor (serpin) protein released by cells of the human foetal retinal pigment epithelium. PEDF promotes the differentiation of retinoblastoma cells into neurons [2]. There are two sets of proteins in the family of serpins. The first group is that of protease inhibitors found in mammals, regulating inflammation, blood coagulation and extracellular matrix modelling. The second group comprises numerous serpins that share structural similarities, but are not believed to inhibit specific proteases; nevertheless, they play significant roles in various cellular processes. PEDF belongs to the second category of serpins. Although it does not have a known target protease, it plays a crucial role in two essential cellular processes, proliferation and survival [3].

Following the investigation into PEDF’s neurotrophic effects, the human PEDF gene was isolated and decoded [4]. It is believed that in the course of evolution, certain non-inhibitory serpins, such as PEDF, have forfeited their capacity for protease inhibition while concurrently acquiring additional characteristics. Notably, PEDF does not undergo the serpin conformational shift that impacts inhibitory serpins when they interact with target proteases [5]. The serpin gene family is believed to have originated over 1.5 billion years ago, predating the divergence of plants and animals [6]. The PEDF region shows synteny in mammals, birds and amphibians, indicating a high level of conservation of this gene cluster throughout a significant period of evolution, and it first appeared in vertebrates. There are significant similarities or homologies when comparing the 5′ sequence with the PEDF gene in human and non-mammalian species [3]. 

The gene for PEDF, a soluble monomeric glycoprotein stemming from the non-inhibitory serpin family [7] (also termed serpin superfamily F member 1 (SERPINF), early population doubling level cDNA-1 (EPC1) and caspin gene [8]), is located on chromosome 17p13.3 and consists of nine exons, which encode a 418-amino-acid protein (NGBI Reference sequence: NG_0281801) [2,9,10,11]. PEDF can be extensively found in various tissues, both in the foetal and adult stages. These tissues include the brain, spinal cord, eye, plasma, bone, prostate, pancreas, heart and lung [12,13]. In regular skin, PEDF levels are elevated, but notably reduced in the initial stages of wound healing [14]. The role of PEDF as an anti-cancer agent, the subject of this review, stands out as one of the most promising prospects among these potential uses [12].

This protein is found in substantial quantities in the cerebral spinal fluid (CSF), potentially secreted by the ependymal cells that line the CSF compartments. PEDF mRNA is detected in many human foetal and adult tissues, and the protein is released in primary cultures of various cell types, including endothelial cells and osteoblasts. In plasma, PEDF is present at concentrations of approximately 100 nM (5 µg/mL), twice that of the required amount to stop abnormal angiogenesis in the eye [15,16].

PEDF’s neuroprotective qualities are at least as effective as those of well-studied factors like brain-derived neurotrophic factor (BDNF) and ciliary neurotrophic factor (CNTF), while its antiangiogenic properties surpass those of other factors such as angiostatin and endostatin [17]. Despite its widespread presence in various tissues, it remains uncertain whether all cell types within a tissue express PEDF or if there are specific spatiotemporal domains of expression. There is no evidence suggesting that PEDF is constantly active in a particular tissue; its broad distribution may represent a starting point for a more intricate pattern of PEDF activation [17].

### 1.2. PEDF Receptors 

Receptors include patatin-like phospholipase domain-containing protein 2 (PNPLA2)/PEDF-R^N^/adipose triglyceride lipase (ATGL), laminin receptor, lipoprotein receptor-related protein 6 (LRP6), plexin domain-containing 1 (PLXDC1), PLXDC2, F1-adenosine-5’-triphosphate synthase (F1 ATP synthase), and vascular endothelial growth factor receptor 2 (VEGFR2) [11,18,19,20]. Under normal circumstances, PEDF receptors create homo-oligomers, and PEDF disrupts these homo-oligomers to trigger receptor activation. Mutations in the intracellular domain can significantly impact receptor functions [21]. The absence of observable abnormal traits in the eye and prostate gland of ATGL knockout mice suggests a partial overlap in phenotypes with PEDF knock-out mice, providing further evidence for the existence of multiple receptors for PEDF [22]. Which receptor is needed depends on the cell or tissue type. For instance, laminin receptor is activated in endothelial cells [23]. PEDF binds to the laminin receptor through a specific region of PEDF, and this interaction is associated with the anti-angiogenic functions of PEDF. Additionally, PEDF acts as a ligand for a third membrane protein, F1 ATP synthase, which is expressed on the surfaces of endothelial and tumour cells [24].

## 2. PEDF in Other Diseases

Over the course of three decades of study, an increasing number of biological roles performed by PEDF have come to light [25], and its roles in some of the diseases within an extensive spectrum are discussed here. PEDF has links to metabolic diseases such as diabetes mellitus (DM) [26]. Increased levels of PEDF in the bloodstream of children suffering from type 2 DM (T2DM) are caused by obesity [27]. It increases after metformin treatment but is not related to insulin resistance, body mass index (BMI) or glycaemic control [28]. 

Lower levels of PEDF have been demonstrated in women with endometriosis [29]. It has been tested as a gene therapy for endometriosis-induced rats, and the outcome was comparable with danazol and more efficient than a placebo [30,31]. PEDF is downregulated in condyloma acuminatum due to angiogenesis augmentation and cell proliferation. It has been found that its corresponding mRNA levels are also lower than normal in such lesions [32]. 

Advanced glycation end products (AGEs) have been demonstrated to play a role in various disorders associated with aging, including cancer. Growing evidence indicates the pathological association of AGEs with aging-related conditions such as cancer, cardiovascular disease, DM, osteoporosis, and Alzheimer’s disease (AD). PEDF exerts an inhibitory effect on AGE-induced proliferation, as well as the expression of VEGF and MMP-9 in breast cancer cells, accomplished through its interaction with the laminin receptor [33].

PEDF enhances the survival of neurons [3]. PEDF decreases during aging and age-related diseases such as age-related macular degeneration (AMD), diabetic retinopathy, Alzheimer’s (AD) and Parkinson’s diseases [34]. PEDF shields retinal neurons from damage caused by light, oxidative stress, and excessive glutamate [17]. The alterations in gene expression observed in senescence are particularly intriguing. PEDF is among the genes that undergo a significant decrease in expression levels as cells age [35]. Therefore, PEDF has the added benefit of safeguarding neurons, which are commonly harmed in vascular diseases of the nervous system.

Previously, heightened levels of PEDF have been suggested as a potential CSF biomarker for AD. There is a significant increase in PEDF levels in the CSF of patients diagnosed with AD, frontotemporal dementia (FTD), and bacterial meningitis. PEDF in the CSF was proposed as a biomarker for AD in earlier research [36]. However, other studies have presented conflicting findings, reporting no change in PEDF levels compared to control patients. Given that PEDF is neuroprotective and antagonises inflammation, it is possible that elevated levels of PEDF in AD patients may occur as a consequence of injury to the brain. In AD patients, the concentration of PEDF in the CSF is proportionate to the levels in the serum [37].

PEDF is recognised for promoting lipolysis in adipose tissue and the liver. Clinical findings indicate a positive association between PEDF and various cardiometabolic risk factors such as BMI and the levels of fasting glucose. T2DM patient serum PEDF levels positively correlate with insulin resistance. In newly diagnosed T2DM patients, insulin administration (either oral or i.v.) leads to a 15% decrease in PEDF levels in the serum. Chen et al. (2016). demonstrated that PEDF reduces angiogenesis, oxidative stress and inflammation in diabetic retinopathy [38]. 

PEDF/VEGF is of utmost importance in the natural regression of infant haemangioma and in the therapeutic efficacy of propranolol [39]. VEGF-induced activation of endothelial cells results in the production of matrix metalloproteinases (MMPs). Within the VEGF family, VEGF-A, VEGF-B, VEGF-C, and VEGF-E stimulate the proliferation of blood vessels by interacting with their specific receptors. Concurrently, VEGF-C and VEGF-D play roles in the process of lymphangiogenesis [40].

The concentration of PEDF as an immunosuppressive is elevated in the tears of patients with dry eye disease (DED). In DED mice, increased PEDF levels were observed in corneal epithelial cells (CECs), but not in the corneal stroma or conjunctiva. Both in vivo and in vitro studies demonstrated that PEDF suppressed the expression of inflammatory cytokines such as IL-1ß, IL-6, TNF-a, and IL-17A, as well as the percentage of Th17 cells in DED [41,42]. Further investigation revealed that PEDF inhibited the phosphorylation of mitogen-activated protein kinase (MAPK) p38 and Jun N-terminal kinase (JNK) in hyperosmotic human CECs. The source of PEDF in CECs was found to be increased in DED, and PEDF played a role in reducing inflammation and regulating the immune response in the development of DED [41]. Interestingly, the results indicated that PEDF levels increased under hyperosmotic conditions, but decreased under cytokine stimulation. Additionally, PEDF was also found to be upregulated in the tears of DED patients [43]. However, previous studies suggested that PEDF expression was downregulated in aged meibomian glands and denervated corneas [44,45]. 

On the other hand, CECs were found to negatively regulate ocular inflammation to maintain homeostasis by secreting factors like PEDF and thrombospondin (TSP)-1, as well as membrane molecules like PD-L1 (B7-H1). TSP-1 was upregulated while PD-L1 was downregulated in DED, indicating that endogenous anti-inflammatory molecules may either increase or decrease in DED [46,47,48]. The increased expression of PEDF was hypothesized to represent a compensatory anti-inflammatory mechanism in DED development [41]. When DED mice were treated with recombinant PEDF (rPEDF), there was a significant prevention of damage to the corneal epithelium [44]. As it is well established that in DED, corneal epithelium damage is primarily caused by inflammation [49], in vivo and in vitro results showed that PEDF inhibited the expression of several pro-inflammatory cytokines (IL-1b, IL-6, and TNF-a) in CECs, hindering the activation of antigen-presenting cells and effector T cells [41].

The expression levels of PEDF were notably lower in squamous cell carcinoma, verruca vulgaris, and psoriasis compared to normal skin. Additionally, the predominant pattern of PEDF expression in normal skin was cytoplasmic, and this differed significantly from the expression patterns observed in psoriasis, squamous cell carcinoma, and verruca vulgaris. PEDF plays a crucial role in various processes, including keratinocyte differentiation, proliferation, skin angiogenesis, and inflammation-related diseases. The observed expression pattern of PEDF may serve as a valuable marker for keratinocyte differentiation [50].

### PEDF Anti-Cancer Functions

Cancer remains a major problem around the world [8]. This is despite the introduction of new forms of therapies clinically. While effective and safe therapies are still lacking, novel diagnostic and prognostic tools have dramatically improved to the extent that cancer as a whole is more treatable. The search for safer and effective therapies has led to the testing of biologicals, PEDF being an example of this. To this end, PEDF employs its anti-cancer effects through various mechanisms. It directly eliminates tumour-activated endothelium (TAE) by using the Fas/FasL intrinsic death pathway and inhibiting the production of cellular-FLICE-like inhibitory protein (c-FLIP), which is a variant of the pro-apoptotic caspase-8 protein [51]. 

Research indicates that PEDF might also engage with degradative enzymes in the extracellular matrix, including matrix metalloproteinase (MMP)-2 and -9, thereby impacting the invasion of tumour cells [52]. Moreover, PEDF reduces the levels of VEGF, hypoxia inducible factor (HIF)-1α and MMP-9, while increasing the expression of TSP-1/2 and angiopoietin-2. As a result, it disrupts the equilibrium between anti- and pro-angiogenic factors [10]. This combination of activities makes PEDF an exceptional and potent candidate for therapeutic use, and its dual capability to trigger the differentiation of cancer cells and hinder the formation of new blood vessels brings extra therapeutic value in the treatment of various malignancies [8,17].

**Figure 1:**
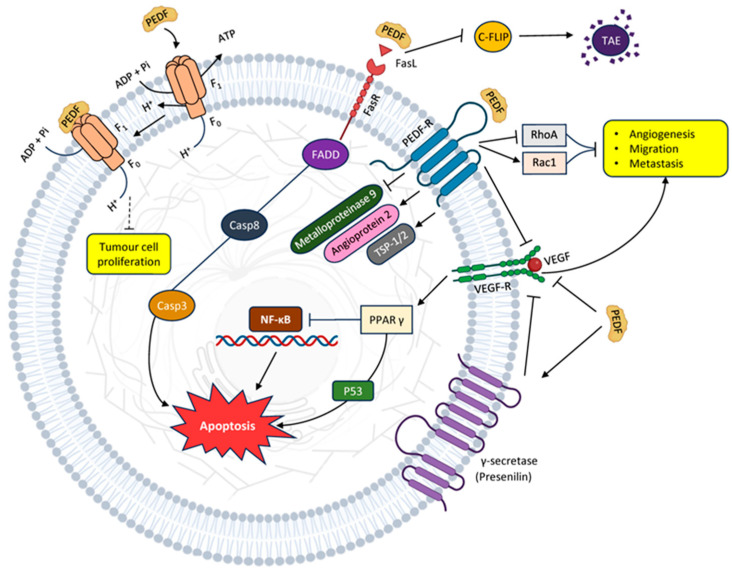
Major known PEDF anti-cancer pathways: inhibition of angiogenesis and proliferation, migration and metastasis leading to apoptosis.

Nuclear factor kappa B (NF-кB) has been identified as the primary intracellular messenger for PEDF. NFκB triggers the production of genes that prevent cell death and promote neurotrophic factors, crucially influencing cell survival, growth, and demise (Figure 1) [17]. 

Inhibiting NF-кB hampers the metastasis of breast cancer to the bone and curbs osteoclastogenesis and osteolysis [53]. NF-кB, a cluster of transcription factors, governs pathways related to inflammation, viability, and cell differentiation in in vitro cell stress models [54]. PEDF blocks NFκB, fostering cellular survival by means of anti-inflammatory and antioxidant effects [55,56]. Conversely, in terms of hindering angiogenesis, PEDF prompts NFκB activity, leading to apoptosis in endothelial cells (Figure 1) [57].

## 3. PEDF Molecular Pathways in Cancer and Role in Cancer Prognosis and Treatment

Palmieri and colleagues carried out a study to examine how PEDF behaves and functions in human endometrial stromal fibroblasts (ESFs) and endometrial cancer cells. The findings demonstrated that PEDF expression and function decrease as cells get older and undergo senescence in vitro. The researchers emphasised that a reduction in PEDF expression due to aging could potentially raise cancer rates in the elderly [58]. PEDF is a potent suppressor of angiogenesis, and more recently, it has been found to limit the processes of tumour development, infiltration, and spread. For instance, in osteosarcoma, elevated expression of PEDF decreases tumour cell proliferation and Matrigel invasion [59]. 

PEDF has both direct and indirect effects on tumour proliferation, effectively suppressing its progression [8]. PEDF exerts its anti-tumour effects through two primary mechanisms. Firstly, it acts directly by promoting reduced cell proliferation and enhanced differentiation. Secondly, it acts indirectly by inhibiting angiogenesis, which is indicative of the phosphorylation status of PEDF [13]. Apart from switching on the expression of proangiogenic factors for its growth and survival, the tumour also has the ability to switch off transcription of antiangiogenic factors [60].

PEDF is believed to demonstrate its antiangiogenic effects through two primary pathways: inducing endothelial cell apoptosis by activating the Fas/Fas-L death pathway and disturbing the delicate equilibrium between pro- and anti-angiogenic factors by suppressing the expression of VEGF [61]. PEDF possesses an asymmetric charge distribution, with a notable accumulation of basic residues at one end and acidic residues at the other. The negatively charged PEDF exhibits robust binding to collagen but does not display neurotrophic effects, instead imparting antiangiogenic properties [52].

When PEDF interacts with tumour cells, it triggers apoptosis through the induction of the Fas-FasL pathway. Additionally, PEDF hampers cell proliferation by reducing the number of cells entering the S-phase, thereby increasing the proportion of cells in the G_0_ resting phase. While PEDF also plays a role in cell differentiation, the exact mechanism underlying this process remains unknown. On an indirect note, PEDF significantly impacts tumour angiogenesis. It achieves this by inducing apoptosis in endothelial cells and reducing the expression of pro-angiogenic factors. Consequently, PEDF disrupts the supply of oxygen and nutrients to the tumour, ultimately leading to the death of tumour cells [13].

PEDF suppresses the growth of retinoblastoma by exerting anti-angiogenic effects. It has been reported that PEDF has a therapeutic role. Retinoblastoma is the most frequent cancer of the eye. In one study, rPEDF prohibited proliferation, and induced apoptosis of endothelial cells, but demonstrated no effects on a human retinoblastoma cell line (SO-Rb50) [62]. The capacity of PEDF to stimulate the differentiation of retinoblastoma cells and other tumour cells originating from neurons was an early indication that PEDF might directly impact tumours and decrease their malignancy. In this context, retinoblastoma cells treated with PEDF exhibit reduced tumorigenicity compared to untreated controls, as demonstrated by delayed tumour formation in rat retinas. Moreover, in the brain, Schwann cells, which naturally secrete PEDF, promote the differentiation of neuroblastoma cells towards a less aggressive phenotype [8].

PEDF controls blood vessel formation and tumour mass of the prostate and pancreas [63]. In vitro, PEDF augments the tumoricidal action of macrophages against prostate cancer cells [64]. PEDF and VEGF have important roles in prostate cancer [65]. They have a reverse correlation with each other in a cancer environment in which decreased levels of PEDF lead to an increase in metastasis and poor prognosis [66]. In pancreatic cells, PEDF is the cause of neuroendocrine differentiation, in addition to preventing tumour angiogenesis [8]. In this cancer, PEDF levels decrease both in the tissue and plasma [7].

PEDF inhibits breast cancer metastasis by downregulating fibronectin. PEDF demonstrated significant suppression of both the proliferation and metastatic spread of breast cancer both in vitro and in vivo [67]. PEDF binds to PEDF-receptor (PEDF-R), thus activating its phospholipase activity. When PEDF-R is located on the cell membrane, its phospholipase A2 (PLA2) active site is positioned near the lipid bilayer, enabling it to utilise lipids as substrates. As testament to this, depending on the levels of omega-3 docosahexaenoic acid (DHA) and omega-6 arachidonic acid (AA) residing in the lipid membranes, PEDF-R can release free DHA or AA. While DHA serves as a precursor for the anti-angiogenic and neuroprotective compound neuroprotectin D1 (NPD1), other metabolites of DHA, such as hydroxy-DHAs, can act on the peroxisome proliferator-activated receptor γ (PPARγ) [8]. 

PEDF downregulates the decoy c-FLIP. Decoy receptors are distinct from death receptors due to their absence of a death domain. Consequently, when they bind to ligands, they cannot trigger the creation of a death-inducing signalling complex (DISC). Instead, they vie with death receptors for ligand attachment, thereby obstructing the process of apoptosis. Additionally, c-FLIP impedes the extrinsic pathway by competing with caspase-8 for Fas-associated death domain (FADD) binding and directly interacts with caspase-8 to render it nonfunctional. In this context, PEDF promotes apoptosis by lowering the levels of an anti-apoptotic inhibitor (Figure 1) [12].

**Figure 2:**
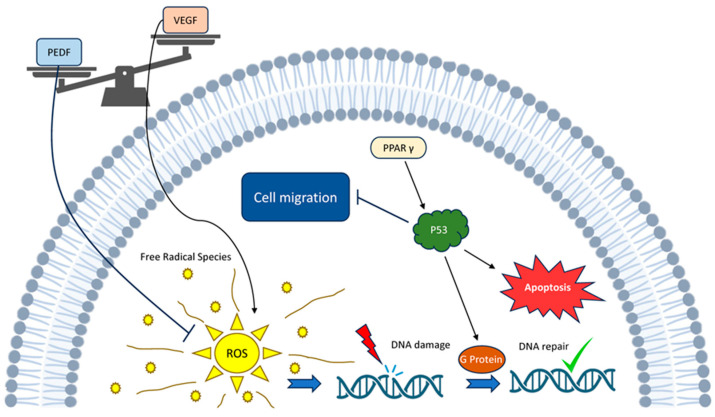
PEDF–p53 pathway interaction: PEDF counteracts with VEGF within a system including interaction between ROS and p53 along with other agents.

The p53 gene is renowned as the “genome guardian”, as a mutation in the p53 gene has been observed in almost all cancer types [12]. Normal and tumour endothelial cells exhibit a strong similarity, sharing numerous markers specific to endothelial cells. Moreover, tumour-derived endothelial cells are distinct in quality from those in normal tissues of the same kind and differ from primary endothelial cultures [68]. Intriguingly, PEDF selectively triggers apoptosis in endothelial cells in actively remodelling blood vessels rather than mature, existing ones [69]. The intersection of PEDF and p53 in such a pathway is naturally intriguing and has already been proposed [70,71]. PEDF carries out its functions by triggering the activation of p53, which then controls the double-strand break repair pathway and initiates the G protein activation pathway (Figure 2) [72]. 

A recent analysis combining multiple studies revealed a significant correlation between low PEDF protein expression in cancer and more advanced cancer progression, as well as markedly poorer survival. The diverse clinical outcomes observed among patients with different levels of PEDF expression underscore its potential as a prognostic indicator [73].

In addition to its significant impact on endothelial cells, PEDF also exerts influence on various other cell types. PEDF can stimulate pericytes to produce platelet-derived growth factor-B (PDGF-B), leading to better pericyte survival which can maintain microvascular homeostasis. In neonatal astrocytes, PEDF stimulates the activation of NF-кB or cyclin AMP-responsive element binding protein (CREB), inducing proinflammatory and immediate-early genes. PEDF has effects on both neurons and neonatal astrocytes. In neurons, PEDF induces the expression of pro-survival genes, such as c-IAP1, c-IAP2, FLIPs, A1/Bfl-1, and MnSOD. Notably, the pro-apoptotic members of the Bcl-2 family, Bax and Bid, remain unaffected by PEDF [74].

p73, a counterpart of p53, emulates the majority of p53’s tumour-suppressing roles [75]. PEDF has been demonstrated to lower VEGF levels in a number of different types of cancer cells [76,77,78,79]. Furthermore, the elevation of PPARγ leads to the upregulation of ρ53 expression, which in turn triggers apoptosis [70]. A substantial cluster of genes regulated by p53 can generate elevated levels of reactive oxygen species (ROS), intensifying the apoptotic response. The outcomes of alterations in intracellular ROS levels prompt diverse responses, with considerable dependence on the specific cell type and the magnitude of ROS. When cells are exposed to heightened ROS levels, oxidative stresses ensue, contributing, among other effects, to the activation of the p53 response. Notably, an observed increase in ROS associated with a p53 deficiency significantly amplifies the oxidation of nuclear DNA and the rate of mutagenesis [80] (Figure 2).

In a retrospective study with 12 children who had lymphangioma cervicofacial lymphangiectomy, scant PEDF staining contrasting with widespread VEGF staining were observed in tumour biopsies. Sections from recurrent disease had increased angiogenesis which correlated with heightened VEGF staining and dampened PEDF staining [81]. 

In an animal study, systemic administration of PEDF in Wilm’s tumour paediatric cases prevented tumour proliferation by targeting tumour vascularisation and tumour cells directly [82]. The same researchers performed a clinical study a few years later and found out PEDF levels were reduced in Wilm’s tumour specimens [83].

PEDF has been associated with tumour invasion, PEDF expression, lipid metabolism, and adipogenesis. PEDF concentration decreased in pancreatic cancers, and its expression was associated with decreased hepatic metastases and better prognosis [84]. This study reported a negative correlation between PEDF and VEGF concentration in human pancreatic cancer. PEDF may prove to be beneficial in the future treatment of cervical cancer, one of the most common and lethal cancers in women [85].

Head and neck squamous cell carcinoma (HNSCC) extensively produces VEGF, which is linked to a more aggressive HNSCC phenotype [86]. VEGF is an angiogenic factor counterpart of PEDF, which has ability to inhibit apoptosis of endothelial cells [87]. The process of angiogenesis relies on maintaining an equilibrium between PEDF and VEGF [88]. Lack of PEDF in cells of nasopharyngeal carcinoma initiates the process of epithelial–mesenchymal transition, leading to metastasis [89].

Melanoma cells prevent the decrease in PEDF in fibroblasts, leading to the development of a cancer-promoting phenotype in cancer-associated fibroblasts [90]. An intriguing discovery was made, in that PEDF phosphomimetic mutants were shown to significantly decrease the development of tumours and curb the spread of tumours in melanoma xenografts within nude mouse models compared to the regular PEDF [91]. Thus, PEDF phosphomimetic mutants may offer more effective results in preventing and impeding metastasis, though this needs more testing [92]. The inhibitory action of PEDF on primary melanoma tumour xenografts is mainly related to suppressing microvessel density (MVD) in the tumour [8]. The overexpression of PEDF through adeno-associated virus-mediated (AAV) gene transfer inhibits the growth of Lewis lung carcinoma (LLC) and suppresses metastasis in a mouse model of colorectal peritoneal carcinomatosis by reducing MVD and increasing tumour cell apoptosis [61,93].

Tissue microarray studies demonstrated a significant decrease in PEDF protein in tamoxifen-resistant/recurrent tumours when compared to primary tumours. In one study, tamoxifen was orally administered at a dose of 1.5 mg per day per mouse for 5 days a week over a span of 21 days, while rPEDF was delivered through intraperitoneal injection at a dosage of 4 mg per kg every 2 days for 21 days. It was observed that the stable expression of PEDF in Michigan Cancer Foundation 7 (MCF-7) cells markedly reduced the levels of oestrogen receptor (ER) α, p^Ser167^ERα, phosphorylated Ak strain transforming (AKT), and the proto-oncogenic receptor tyrosine kinase rearranged during transfection (RET) [16]. 

Thus, various studies have trialled PEDF against cancer, though not clinically as yet. The major studies performed to date are summarised in Table 1.

### Inflammation

Inflammation can lead to cancer initiation and progression. Tumour necrosis factor (TNF)-α, as the most pleiotropic cytokine, is an important factor in the control of inflammation [94,95]. There is also an interaction between cytokine signalling and non-coding RNAs in the pathobiology of HNSCC [86].

Some studies have discovered that PEDF has the ability to dampen inflammation in vivo by controlling the NF-кB signalling pathway, thus mitigating the elevated cellular levels of ROS induced by angiotensin II (Ang) [88,96]. PEDF decreases tumour necrosis factor (TNF)-α in breast cancer [97]. On the other hand, TNF-α and other inflammatory cytokines such as interleukin (IL-β) and IL-6 inhibit PEDF expression [41,98]. Additionally, ROSs are triggered as a downstream effect of VEGFR2 activation [99].

Pancreatic cancer is distinguished by a pronounced fibro-inflammatory response known to contribute to cancer progression. Previous studies have highlighted the potent tumour-suppressive effects of PEDF in this cancer type, but the underlying mechanisms are still not well known. Nevertheless, PEDF expression is reduced in clinical pancreatic samples compared to benign matched tissues. Furthermore, human tumour specimens that lack PEDF exhibit increased inflammation and fibrosis [100]. 

Knockout of PEDF in mice increased inflammation and fibrosis induced by cerulein, and similarly intensified these events in the presence of oncogenic Kirsten rat sarcoma viral oncogene homolog (KRAS). rPEDF inhibited macrophage migration, macrophage-induced tumour cell proliferation, and the production of pro-fibrotic and pro-inflammatory factors. One indication of PEDF’s ability to reduce fibrosis was the reduced deposition of collagen I and expression of TGF-b in pancreatic stellate cells. PEDF dysregulated pancreatic cancer progression by reducing both fibrosis and inflammation. Thus, PEDF has been proposed to be a potential therapeutic test agent for pancreatic cancer [100]. 

Clinical pancreatic tumours void of PEDF staining show increased inflammation and fibrosis within the tumour sample. Such a finding has also been seen in pancreatic tumour animal models. It is important to note that PEDF downregulation is not unique to pancreatic cancer, with restored PEDF linked to decreased tumour growth and improved animal survival in tumour models [100].

The phenomenon of attenuation of inflammation by PEDF has also been noted in metabolic syndrome [101]. In diabetic retinopathy, for example, the inhibition of PEDF activity results in heightened pro-inflammatory cytokine production and macrophage recruitment [102,103,104]. The administration of PEDF inhibited macrophage migration. There are contradictory reports indicating that PEDF has pro-inflammatory effects [100,105]. However, this depends on the environment, as PEDF has been shown to recruit macrophages but only for the purpose of targeting tumour cells [106], so the overall result was beneficial from the therapeutic perspective. In prostate cancer, PEDF attenuates NF-кB-mediated upregulation of IL8, a pro-inflammatory cytokine crucial in cancer progression, particularly pancreatic cancer [107,108,109].

**Table 1:** A summary of major studies on cancer with PEDF.

Studied Features	Method/Type	Results	References
Effect of PEDF + RT on NPC	In vivo	↑Anti-tumour effects by ↑VEGF	[110]
Effect of ↑PEDF on NPC	In vitro	Inhibit lymphangiogenesis	[25]
Effect of ↓PEDF on NPC	In vitro	EMT and metastasis	[89]
Cervicofacial lymphangioma tumour specimens	Clinical	↓PEDF and ↑VEGF	[81]
Fresh and frozen Wilms’ tumour samples	Clinical	↓PEDF	[83]
Gastric cancer	Clinical	↑PEDF, ↑VEGF, ↓TNF-α in serum	[111]
TNBC	Clinical	↓PEDF, ↑NF-кB	[97]
Metastatic breast cancer	In vitro	↓PEDF	[95]
Colorectal cancer	In vitro and in vivo	↓PEDF	[9,112]
Castration refractory prostate cancer	↑PEDF in vitro	↑Anti-tumour effect of chemotherapy	[113]
Osteosarcoma	↑PEDF in vitro	↓Primary and metastatic tumours	[114]
Breast cancer	Clinical	↑PEDF ~ ↓MVD	[115]

***Key:*** *EMT*, epithelial-mesenchymal transition; *NF-кB*, nuclear factor-kappa B; *NPC*, nasopharyngeal carcinoma; *TNBC*, triple negative breast cancer; *TNF-α*, tumour necrosis factor-α; *RT*, radiotherapy; *MVD,* microvessel density; *VEGF*, vascular endothelial growth factor.

## 4. Chemotherapy of Cancer in Combination with PEDF

PEDF combined with low-dose chemotherapy (CT) can provide a promising option as a new therapeutic alternative for castration-refractory prostate cancer, because since the advent of multi-agent chemotherapy regimens in the 1970s, there has not been a substantial improvement in patient prognosis [113]. When paired with specific anti-angiogenic agents, low-dose chemotherapy has demonstrated considerable promise. As PEDF possesses strong antiangiogenic properties, its examination in conjunction with the chemotherapeutic doxorubicin, when applied to a chemoresistant osteosarcoma tumour, enhances the tumour’s responsiveness to treatment [116,117]. Other researchers have focused on interleukin (IL)-8 as a crucial regulator of resistance to traditional chemotherapy medicines such as docetaxel (DTX/Taxotere) [113].

In one study [118], PEDF’s inclusion led to a decrease in the IC_50_ (half-maximal inhibitory concentration) of oxaliplatin and irinotecan in a panel of tumour cell lines. Treatment of animals with tumour xenografts with PEDF carboxy-terminus peptides decreased chemoresistance against conventional CTs, and decreased metastasis as has been seen with the full protein in other cancers such as osteosarcoma [59,114]. The PEDF signalling pathway holds promise as a prospective therapeutic tool for adjuvant therapy, potentially lowering IC_50_ doses, minimising adverse effects, and reducing treatment costs. Moreover, this pathway may play a role in extending the time to relapse in patients, diminishing tumorigenicity, and limiting the ability to generate metastases [118].

Another study found that TSP-1 and PEDF reduced MVD in a xenograft model of colon adenocarcinoma. These factors also heightened macrophage infiltration and response to metronomic cyclophosphamide (CPA) administration. PEDF increased the anti-metastatic effects of treatment, but did not activate the innate anti-tumour immunity induced by metronomic CPA [119]. 

## 5. Radiotherapy of Cancer in Combination with PEDF

RT with photon beams (not proton beams) targets the cytoplasm to produce ROS—that is, free radicals which diffuse into the nucleolus to damage the DNA in nanoseconds. This effect can be therapeutic in damaging the reproductive integrity of the cancer cells, but also detrimental in damaging normal tissues [120].

RT harms endothelial cells, leading to fibrosis. This can result in tissues becoming hypoxic, hypocellular and hypovascular, which, in turn, can lead to necrosis and ulceration [121]. This means that cancer cells with higher levels of HIF-1α are more resistant to RT [122]. ROSs are eventually produced due to DNA damage through ionising radiation, and they play important roles in the induction of cell death pathways (Figure 2) [123,124]. Oxidised low-density lipoprotein (ox-LDL) causes a decrease in PEDF levels by increasing the production of ROS. However, D-4F (an apolipoprotein A-I mimetic peptide) can shield endothelial cells from injury induced by ox-LDL by averting the decrease in PEDF levels [88]. On the other hand, PEDF diminishes ROS generation in endothelial cells exposed to oxidative stress caused by hyperglycaemia, ultimately resulting in apoptosis, which serves as an anticancer mechanism [96,125]. Redox homeostasis pertains to maintaining equilibrium between the generation of ROS and reactive nitrogen species (RNS) [126].

Various forms of oxidative harm to DNA exist. Different stressors can cause oxidative DNA damage. SSB and DSB are the critical mechanisms of expression of radiation damage, namely the loss of reproductive integrity, misrepair and repair. The main cause of base mismatches in DNA arises from replication stress, while single-strand breaks (SSBs) and, to a lesser extent, double-strand breaks (DSBs) are the chief ones. Both SSBs and DSBs, along with interstrand crosslinks, can be induced by ionising radiation and chemotherapy. UV radiation-induced DNA damage leads to the formation of substantial DNA adducts [126]. Increasing the levels of PEDF in highly metastatic rectal carcinoma cells resulted in heightened sensitivity to radiation and inhibited their ability to migrate and invade [72]. Recent studies have shown that combining PEDF with radiotherapy enhances the effectiveness of each agent in an animal model of nasopharyngeal carcinoma [110]. 

A study illustrated that the use of DC101, a blocker of vascular endothelial growth factor receptor 2, created a period referred to as a “normalisation window” and decreased the levels of intratumoural hypoxia in U87 glioma xenografts [127]. Furthermore, the researchers discovered that when radiation therapy was combined with DC101 during this “normalisation window”, it produced the most favourable antitumor results. Another investigation demonstrated that bevacizumab increased intratumoural oxygen levels and amplified the effectiveness of ionising radiation in treating gliomas [128]. In this research, it was discovered that PEDF by itself did not trigger tumour cell apoptosis, a result that contrasts with an earlier study. Nonetheless, this incongruent outcome could be attributed to the in vivo test in this research and the utilisation of distinct cancer cell lines [129]. The results were in line with earlier research. It was observed that the radiation applied during the period of “normalisation” induced by PEDF treatment had the most substantial impact on restraining the growth of LLC [129]. The heightened sensitivity to radiation caused by PEDF could be linked to improved oxygen levels. There was a decrease in tumour hypoxia during this “normalisation window”. 

As oxygen significantly enhances the effectiveness of ionizing radiation, a temporary boost in oxygen levels would enhance the outcomes of radiation therapy [130]. In summary, this study showed that PEDF created a “vascular normalisation window” between the 3rd and 7th day following treatment, leading to a notable improvement in the anti-tumour effects of radiation in an LLC allograft model. As a result, the combination of PEDF and radiation administered at the appropriate timing substantially suppresses the growth of lung cancer [129]. Remarkably, phosphomimetic variants of PEDF exhibit antitumor activity comparable to the established anti-angiogenic agent bevacizumab but operate independently of VEGF, without affecting VEGFA mRNA levels or VEGF receptor 2 phosphorylation [131]. In another study, there was no significant difference between the efficacy of topical PEDF and bevacizumab in the treatment of corneal neovascularization [132].

## 6. Cancer Biomarkers and Their Relationships to PEDF in Cancer 

Hypoxia refers to an abnormal level of oxygen tension, commonly observed in a majority of malignant tumours. Tumour-induced hypoxia leads to advanced yet dysfunctional vascularisation and the transition from epithelial to mesenchymal phenotype, enhancing metastatic capacity. This transition has the dual effect of the above plus contributing to CT resistance, involving pathways such as HIF, phosphoinositide 3-kinase (PI3K), MAPK, and NF-кB, which interact with each other, creating positive and negative feedback loops that either enhance or diminish the effects of hypoxia [120].

Brief exposure to hypoxia enables cells to survive adverse conditions by activating autophagy, a cellular adaptation involving a decrease in oxidative metabolism. Conversely, cycling hypoxia has been demonstrated to increase the production of ROS, contributing to the survival and progression of tumour cells. Both short- and long-term hypoxia increase cancer cell radioresistance in cell culture and in vivo. When tumours advance to a more aggressive stage, acute hypoxia usually sets in, due to the inability of the blood supply to keep up with the oxygen needs of the tumour cells, usually culminating in metastasis [120]. 

Tumour hypoxia arises from uncontrolled cell proliferation, altered metabolism, and abnormal tumour blood vessels, leading to reduced oxygen and nutrient transport. Despite being lethal for normal cells and most tumour cells, a small sample of tumour cells within a tumour can develop resistance to hypoxia and survive. Unfortunately for cancer clinicians and patients, these hypoxia-resistant cells also develop an ability to withstand cytotoxicity due to CT and RT. Therapy resistance due to hypoxia has been studied for the past 6 decades, and is considered a significant feature in solid tumours, correlating with a poor prognosis for cancer patients [120].

Warburg was the first to note changes in the metabolism of cancer cells, coining the term “Warburg Effect” to describe their heightened reliance on glycolysis, even in the presence of oxygen, a phenomenon known as aerobic glycolysis. The precise significance of these metabolic alterations in terms of cell growth and the development of cancer is an ongoing focus of therapeutic research. The tumour’s capacity to control different biosynthetic pathways when facing metabolic challenges could offer a promising avenue for the discovery of new therapeutic targets [133].

ROS is a result of metabolic processes, and reduced ATP levels are linked to metabolic strain caused by lack of nutrients or low oxygen. This situation can worsen the stress due to the buildup of ROS. Research indicates that ROS generation in nearby cancer cells prompts the reverse Warburg effect in cancer-related fibroblasts. This effect reduces caveolin-1 (Cav-1), raises nitric oxide (NO) levels, and leads to mitochondrial dysfunction and oxidative stress. Interestingly, this outcome was not replicated in astrocytes during the study. Therefore, it was inferred that only transformed cells respond to ROS-triggered autophagic cell death, which means that ROS may be contribute to the apoptosis of tumour cells [64,134]. Experimental and clinical research has recognised PEDF as a factor that plays a dual role in the development of metabolic syndrome, serving both as a contributing factor and a counter-regulatory agent [101].

As mentioned above, PEDF is a serine protease inhibitor protein with various biological properties. It is emerging as a novel metabolic regulatory protein with a role in insulin resistance. Insulin resistance is a central factor in the pathogenesis of metabolic disorders such as obesity, T2DM, polycystic ovarian disease, and metabolic syndrome, and PEDF is associated with these conditions. PEDF administered to animals induces insulin resistance, while its removal improves insulin sensitivity. Inflammation, the mobilisation of lipolytic free fatty acids, and mitochondrial dysfunction are some of the proposed mechanisms of PEDF-mediated insulin resistance [135].

Biology markers for adhesion, growth and migration include Ki67, Ras homolog family member (Rho) A, cell division control protein (Cdc) 42, Rak, membrane type 1 matrix metalloproteinase (MT1-MMP) [136]. p53 has been extensively investigated as a potential biomarker due to its significant role in the development and treatment of tumours, making it the focus of more studies than any other gene [137].

Within the animal domain, there are three Rho isoforms—RhoA, RhoB, and RhoC—which share an 85% amino acid sequence identity, encompassing Rho, Rac, and Cdc42, and which play pivotal roles in shaping cell structure and facilitating movement. Cdc42 contributes to filopodia generation, while RhoA is instrumental in the assembly of focal adhesions and the formation of stress fibres [99,138]. Interestingly, as of now, no mutations have been identified in Rho proteins. The sole exception within the Rho family of small GTPases is RhoH, which has been reported to undergo genetic alterations in non-Hodgkin’s lymphomas and multiple myeloma [138]. The expression of RhoC in vivo showed a correlation with advanced clinical stage and lymph node metastases in both the overall patient cohort and in small primary tumours (T1 and T2). This research represents the initial investigation into the expression of the RhoC GTPase protein in HNSCC and normal squamous epithelium [139]. Rak is a 54 kDa tyrosine kinase which belongs to the Src kinases family and acts as a tumour suppressor by controlling phosphatase and tensin homolog deleted on chromosome 10 (PTEN) protein function [140].

Metabolic markers in OPSCC cell lines include insulin, insulin-receptor, AMP-activated protein kinase (AMPK), P13K, and Ak strain transforming (AKT) [136]. Akt, also recognized as PKB, serves as a signalling mediator downstream of PI3K and holds significant relevance in the regulation of endothelial cell biology influenced by VEGF [99]. Aldo-keto reductase family 1 member (AKR1C)3 serves as both a biomarker and a target suitable for drug intervention in oropharyngeal tumours [141]. Protein kinase B or Akt (PKB/Akt) represents a serine/threonine kinase. Within mammals, it consists of three closely related variants: PKBa (Akt1), PKBb (Akt2), and PKBg (Akt3). The activation of PKB/Akt occurs in response to various stimuli like hormones, growth factors, and components of the extracellular matrix when cells are exposed to them [142].

The PI3K/AKT signalling pathway is crucial in the development of tumours, overseeing vital cellular processes such as survival, proliferation, and metabolism. Mutations in PIK3CA and the activation of AKT through phosphorylation (pAKT) are frequently observed in various cancers, with breast cancer exhibiting particularly high occurrences. Notably, the presence of pAKT has been recognised as a predictive factor for the effectiveness of paclitaxel CT in breast cancer with lymph node involvement [143].

The Rac1 protein has the potential to be seen as a possible biomarker for radiation resistance in HNSCCs. Additionally, it could be a viable target for therapeutic strategies to treat recurrences of HNSCCs, both locally and at distant sites [144]. ROS also function as regulatory factors, balancing the antagonistic relationship between Rac-1 and RhoA [138]. A new 7-CRL signature has been introduced as an innovative biomarker for forecasting the prognosis of HNSCC. Long non-coding RNAs (LncRNAs) hold promise in both the diagnosis and treatment of HNSCC [145]. Rac and Cdc42 play distinct roles in the regulation of cellular processes; Rac is involved in membrane ruffling, while Cdc42 is associated with filopodium formation [138]. Some of the major biomarkers in cancer that have been associated with PEDF activity are summarised in Table 2.

## 7. Molecular and Gene Therapies with PEDF in Cancer

There are several anti-angiogenic factors in clinical use including VEGF inhibitors, such as bevacizumab (Avastin), tyrosine kinase inhibitors (TKIs), such as sunitinib, thalidomide, and endostatin, and fibroblast growth factor inhibitors (FGFIs) [153]. Research has shown that PEDF is among the most potent physiological and pathological anti-angiogenic factors, with both direct and indirect oppressive effects on tumour proliferation, though this remains to be tested clinically in the context of therapy.

The domain of gene therapy has a history spanning two decades, tracing its roots in the late 1980s when gene manipulation in mammalian cells was first explored [154,155,156]. Around the year 2000, gene therapy achieved its initial breakthroughs, particularly in the highly specialised domain of addressing severe inherited T-cell immune deficiencies through ex vivo gene transfer into hematopoietic stem (HS) or progenitor cells (C) [157]. The possible healing benefits of PEDF have been assessed in lentiviral gene transfer models. Introducing PEDF into human pancreatic cancer cells through this method has led to the suppression of tumour development in mouse models, regardless of whether the gene transfer was performed under the skin or into the abdominal cavity [158].

The PEDF gene (*PEDF*) loaded in poly lactic-co-glycolic acid (PLGA) nanoparticles has been utilised for the treatment of colon carcinoma. A study found for the first time that the PEDF gene loaded in PLGA nanoparticles could have efficiently prohibit proliferation of CT26 tumours [159]. Previous demonstrations indicate that when rPEDF is administered systematically, it leads to tumour regression through a targeted impact on both the tumour itself and its blood vessels. This same effect is observed when using a viral vector to administer PEDF [160,161,162].

Expressing the PEDF gene through AAV could be a proactive method to impede cancer growth. As a case in point, treatment with AAV-PEDF could be useful in lung cancer treatment [61]. Nonetheless, gene therapy encounters drawbacks during clinical trials due to the absence of suitable delivery mechanisms or vectors [163].

Colorectal cancer (CRC) is a significant contributor to global cancer-related fatalities, with metastasis being a primary cause of mortality in CRC patients. Emerging research highlights the ability of the PEDF protein to impede tumour progression through antiangiogenic and pro-apoptotic mechanisms. In one study [164], a PEDF plasmid DNA-loaded liposome formulation (with an iRGD peptide) was used to show tumour cell-selective activity, including the inhibition of invasion, migration and increased apoptosis of CRC in culture [164]. Additionally, these liposomes reduced metastatic tumour nodules in the lungs and extended the survival time in a mouse model of metastatic CRC.

Insufficient gene delivery to tumour sites has contributed to the suboptimal outcomes observed in clinical trials of cancer gene therapy. Despite being the most active and promising field in gene therapy, previous successes in animal models have not translated effectively to human trials, partly due to the challenge of achieving adequate gene delivery to tumour locations. In this regard, the injection of human mesenchymal stem cells (hMSC) expressing PEDF had a notable impact on both the growth of primary liver tumours and the occurrence of pulmonary metastases. Additionally, this approach led to a moderate increase in systemic levels of human PEDF. Histological analysis of primary liver tumours revealed lower MVD in mice treated with hMSCs-PEDF compared to control mice in the treatment of hepatocellular carcinoma (HCC) [165].

Directing genetic medicine specifically to tumours not only prevents adverse effects on normal cells but also optimises the dosage delivered to the cancerous cells. Lipoplexes enhance the intracellular delivery of oligonucleotides. However, achieving optimal delivery of lipoplexes to in vivo target sites like tumours faces various challenges. Other macromolecular carriers, such as nanospheres or microspheres, can also be utilised to target oligonucleotides, and these carriers have demonstrated clinical promise in other therapeutic modalities such as RT. Combining the favourable characteristics of different particles into a single carrier has the potential to enhance the delivery of various classes of potential therapeutic agents to tumours [166].

The scarcity of efficient PEDF-DNA delivery vectors is attributed to immune responses and inefficiency [164]. Clinical trials have demonstrated encouraging potential for nanotechnology-based gene therapy in cancer treatment [167]. Cancer metastasis, a significant factor in global cancer-related deaths, including CRC, remains a formidable challenge [168,169].

## 8. Contradictory Findings

As previously discussed, PEDF mainly has a protective role against cancer based on numerous preclinical and clinical studies. However, its negative deteriorating effect has been revealed by some studies. PEDF facilitates the spread of tumours in HCCs by interacting with the laminin receptor. When PEDF is overexpressed, it intensifies the aggressive characteristics of HCC cells both in vitro and in vivo. Conversely, suppressing the expression of PEDF results in decreased migration and invasion of HCC cells [170].

Glioblastoma multiforme (GBM) stands out as the most highly aggressive malignant primary brain tumour. In primary glioblastomas, the presence of the active epidermal growth factor receptor (EGFR) vIII mutation is common, leading to a persistently active EGFR. These tumours secrete PEDF, with EGFRvIII frequently found in the glioblastoma cells [148]. The presence of PEDF is linked to a favourable outlook and is connected to genes related to EMT in invasive ductal breast cancer, although the expression of PEDF showed a strong connection with tumour size. A positive relationship between PEDF and the expression of E-cadherin, vimentin, Snail, and NF-кB was found [171].

PEDF plays a role in promoting the growth and migration of human OPSCCs. However, the precise mechanisms by which PEDF operates in cancer development are subject to debate. While PEDF exhibits anti-tumour effects in certain cancer types, such as pancreatic, melanoma, and ovarian cancers, it behaves differently in other cancers. In some cases, PEDF levels surpass those found in normal tissues. PEDF has been identified in HCCs, where its levels are elevated compared to normal precancerous tissues. The secretion of PEDF is also higher in HC patients compared to normal controls, suggesting the potential use of serum PEDF as a biomarker for diagnosing HC [172].

Finally, it was found that some of the gathered macrophages exhibited TNF-α expression, and the application of TNF-α resulted in the reduction in both PEDF protein and mRNA expression in prostate AT-1 tumour cells in vitro and in the rat ventral prostate in vivo. PEDF seems to exert diverse effects on prostate tumours, acting to inhibit angiogenesis and metastasis, while simultaneously inducing the accumulation of macrophages. The accrual of macrophages may have a dual impact, potentially inhibiting tumour growth, yet also suppressing PEDF and promoting lymphangiogenesis, ultimately contributing to tumour growth enhancement [173].

Consequently, further investigation is necessary to fully understand the function of PEDF and its underlying mechanisms in various cancer types before it can be confidently employed as a diagnostic or prognostic biomarker or as a component of cancer treatment strategies [172]. At present, no clinical trials are underway for this protein in the context of cancer therapy.

## 9. Conclusions

PEDF has advantages over conventional therapeutics in terms of being naturally found in the body and having high potency compared to other angiogenic factors. Anti-angiogenic treatments must be carefully tailored to target newly formed blood vessels while sparing existing ones in order to minimise unwanted side effects such as normal blood vessel collapse, bleeding, and inappropriate tissue matrix degradation. PEDF adheres to these criteria by exclusively inhibiting the growth of new blood vessels, exhibiting no harmful effects on mature vessels, and regulating vascular integrity [174].

One benefit of employing natural anti-angiogenic molecules like PEDF to combat abnormal blood vessel growth is that they are not expected to activate drug-resistant genes. This makes it promising for long-term angiogenesis therapy [174]. Natural anti-angiogenesis factors can also effectively prevent pathological vasculature without causing damage to the existing vascular network [69]. Furthermore, natural anti-angiogenic molecules are well tolerated by the body and are unlikely to trigger an immune response or produce the toxic side effects associated with synthetic inhibitors [174].

Therefore, PEDF offers an additional advantage by preserving neurons, which are frequently damaged in nervous system vascular diseases. Its dual ability to induce cancer cell differentiation and impede angiogenesis provides added therapeutic value in treating a wide range of malignancies. It therefore could become an atypical and potent contender as a therapeutic agent [10].

The fact that PEDF is naturally produced and widely distributed throughout the body reduces the likelihood of adverse side effects or the development of drug resistance, unlike synthetic agents. However, it is important to note that we have limited knowledge about PEDF’s overall physiological role in the human body, necessitating further investigation before any clinical trials can begin [16].

Notably, no adverse effects resulting from use of PEDF in vivo have been reported in the literature. There are studies highlighting the possible use of PEDF as a prognostic factor in certain types of cancer. Thus, with the initial determination of PEDF levels, tailoring treatments based on this information may improve therapeutic outcomes in cancer patients [8]. However, there is still much work to be done in this area.

## Figures and Tables

**Table 2:** Major cancer biomarkers related to PEDF.

Biomarker (Metabolic/Metastatic)	Functions	References
AMPK	Glucose metabolism	[146]
MT1-MMP	Adhesion and migration	[147]
PTEN	Tumour suppressor	[140]
AKT, P13K	Migration	[143]
Ki67	Viability and proliferation	[148]
RhoA	Cell migration	[149]
p38, ρ73	Migration and colony formation	[112]
p53	Cell fate via ↑PEDF	[150]
AKR1C3	↓Cisplatin action	[141]
Glutamate	Metabolism	[151,152]
LncRNA	Diagnostic and prognostic	[145]
Cdc42	Migration and adhesion	[99]

***Abbreviations***: *AKR1C3*: aldo-keto reductase family 1 member; *AKT*: Ak strain transforming; *AMPK*: AMP-activated protein kinase; *LncRNA*: long non-coding RNA; *MT1-MMP*: membrane type 1 matrix metalloproteinase; *P13K*: phosphoinositide 3-kinase; *PTEN*: phosphatase and tensin homolog deleted on chromosome 10; *RhoA*: Ras homolog family member.

## Abbreviations

*AAV*: adeno-associated virus; *AD*: Alzheimer’s disease; *AGEs*: advanced glycation end products; *AKR1C3*: Aldo-keto reductase family 1 member 3; *AKT*: Ak strain transforming; *AMD*: age-related macular degeneration; *AMPK*: AMP-activated protein kinase; *AngII*: angiotensin II; *ATGL*: adipose triglyceride lipase; *ATP*: adenosine triphosphate; *BDNF*: brain-derived neurotrophic factor; *BMI*: body mass index; *Cav-1*: caveolin-1; *Cdc42*: cell division control protein 42; *CECs*: corneal epithelial cells; *c-FLIP*: cellular-FLICE-like inhibitory protein; *CNTF*: ciliary neurotrophic factor; *CPA*: cyclophosphamide; *CRC*: colorectal cancer; *CREB*: responsive element binding protein; *CSF*: cerebrospinal fluid; *CT*: chemotherapy; *DED*: dry eye disease; *DISC*: death-inducing signalling complex; *DSB*: double-strand breaks; *DTX*: docetaxel/taxotere; *EGFR*: epidermal growth factor receptor; *EMT*: epithelial-mesenchymal transition; *EPC1*: early population doubling level cDNA-1; *ER*: estrogen receptor; *ESF*: endometrial stromal fibroblasts; *FADD*: Fas-associated death domain; *F1-ATP synthase*: F1-adenosine-5’-triphosphate synthase; *FGFI*: fibroblast growth factor inhibitor; *FTD*: frontotemporal dementia; *GBM*: glioblastoma multiforme; *HC*: hepatocellular carcinoma; *HCC*: hepatocellular carcinoma; *HIF-1α*: hypoxia inducible factor-1α; *HPV*: human papilloma virus; *hMSC*: human mesenchymal stem cell; *HNSCC*: head and neck squamous cell carcinoma; *IC50*: half-maximal inhibitory concentration; *IL*: interleukin; *JNK*: Jun N-terminal kinase; *KRAS*: Kirsten rat sarcoma viral oncogene homolog; *LLC*: Lewis lung carcinoma; *LOX*: lipoxygenase; *LRP6*: lipoprotein receptor-related protein 6; *LncRNA*: long non-coding RNA; *MAPK*: mitogen-activated protein kinase; *MCF*: Michigan Cancer Foundation; *MT1-MMP*: membrane type 1 matrix metalloproteinase; *MVD*: microvessel density; *NF-кB*: nuclear factor- kappa B; *NHEK*: normal human epidermal keratinocyte; *NO*: nitric oxide; *NPC*: nasopharyngeal carcinoma; *NPD1*: neuroprotectin D1; *OPSCC*: oropharyngeal squamous cell carcinoma; *OS*: overall survival; *Ox-LDL*: oxidised-low density lipoprotein; *PEDF-R*: pigment epithelium-derived factor-receptor; *PFS*: progression-free survival; *PIK3*: phosphoinositide 3-kinase; *PKB*: protein kinase B; PLA2: *PLGA*: poly lactic-co-glycolic acid; *PLXDC1*: plexin domain-containing 1; *PNPLA2*: patatin-like phospholipase domain-containing protein 2; *PTEN*: phosphatase and tensin homolog deleted on chromosome 10; *RhoA*: Ras homolog family member; *RPC*: retinal progenitor cell; *rPEDF*: recombinant PEDF; *RNS*: reactive nitrogen species; *ROS*: reactive oxygen species; *RPE*: retinal pigment epithelium; *RT*: radiotherapy; *SERPINF1*: serpin superfamily F member 1; *SSB*: single-strand break; *T2DM*: type 2 diabetes mellitus; *TKI*: tyrosine kinase inhibitor; *TNF-α*: tumour necrosis factor-α; *TSP-1/2*: thrombospondin-1/2; *VEGFR2*: vascular endothelial growth factor receptor 2.
